# Increased PDGFR-beta and VEGFR-2 protein levels are associated with resistance to platinum-based chemotherapy and adverse outcome of ovarian cancer patients

**DOI:** 10.18632/oncotarget.18415

**Published:** 2017-06-08

**Authors:** Stefanie Avril, Yasemin Dincer, Katharina Malinowsky, Claudia Wolff, Sibylle Gündisch, Alexander Hapfelmeier, Melanie Boxberg, Holger Bronger, Karl-Friedrich Becker, Barbara Schmalfeldt

**Affiliations:** ^1^ Institute of Pathology, Technische Universität München, Munich, Germany; ^2^ Institute of Medical Statistics and Epidemiology, Technische Universität München, Munich, Germany; ^3^ Department of Obstetrics and Gynecology, Technische Universität München, Munich, Germany; ^4^ Current address: Department of Pathology, Case Western Reserve University School of Medicine, University Hospitals Cleveland Medical Center and Case Comprehensive Cancer Center, Cleveland, Ohio, United States; ^5^ Current address: Department of Gynecology, University Medical Center Hamburg-Eppendorf, Hamburg, Germany

**Keywords:** phosphoproteomics, reverse phase protein array (RPPA), ovarian cancer, platinum chemotherapy resistance, response prediction

## Abstract

Despite frequent initial response rates of epithelial ovarian cancer to platinum-based chemotherapy the majority of patients develop drug resistance. Our aim was to evaluate differential expression of signaling-pathway proteins in platinum-sensitive versus platinum-resistant primary epithelial ovarian cancer specimens to identify predictive biomarkers for treatment response.

192 patients were studied comprising of independent training (*n* = 89) and validation (*n* = 103) cohorts. Full-length proteins were extracted from paraffin-embedded samples including multiple regions per tumor to account for intratumoral heterogeneity. Quantitative reverse-phase-protein-arrays were used to analyze protein and phospho-protein levels of 41 signaling molecules including growth-factor receptors, AKT and MAPK signaling pathways as well as angiogenesis and cell-adhesion.

Platinum-resistant ovarian cancers (56/192) demonstrated significantly higher intratumoral levels of the angiogenesis-associated growth-factor receptors PDGFR-beta and VEGFR2 compared to platinum-sensitive tumors. In addition, patients with high PDGFR-beta expression had significantly shorter overall and progression-free survival (HR 3.6 and 2.4; *p* < 0.001). The prognostic value of PDGFR-beta and VEGFR2 was confirmed in publicly available microarray-datasets.

High intratumoral levels of the angiogenesis-related growth-factor receptors PDGFR-beta and VEGFR2 might serve as novel predictive biomarkers to identify primary resistance to platinum-based chemotherapy. Those ovarian cancer patients might particularly benefit from additional anti-vascular therapy including anti-VEGF antibody or receptor tyrosine-kinase-inhibitor therapy.

## INTRODUCTION

Epithelial ovarian cancer is the second most common gynecologic malignancy and is characterized by the highest mortality of all gynecological cancers [1]. Ovarian cancer is often diagnosed in advanced stages of disease, limiting the prognosis of patients. Standard treatment consists of cytoreductive surgery followed by systemic platinum and taxane-based chemotherapy [2]. Although most women initially respond to chemotherapy, the majority will relapse after first-line treatment and ultimately develop resistance to platinum-based chemotherapy [3]. The development of platinum resistance contributes to the poor outcome of advanced stage ovarian cancer patients [4–6]. Additional biomarkers would be helpful to better predict treatment response and identify potential novel therapeutic targets.

The assessment of cell signaling proteins offers the opportunity to identify potential new drug targets as well as to predict response to treatment and aid in individualized treatment decisions [7]. A number of cell signaling pathways have been previously identified as potential predictive biomarkers and/or therapeutic targets. These include angiogenesis-related and other growth factors and their receptors, VEGF, VEGFR, PDGFR, EGFR or HER2, PI3K, AKT/ mTOR-pathway, and MAP-Kinase pathway [8–11], as well as their activated phosphorylated forms.

Reverse phase protein arrays (RPPA) allow the reproducible and quantitative analysis of large numbers of target proteins and samples under the same experimental conditions, when conventional immunoblot methodology is not suitable [12]. RPPA technology has widely demonstrated its utility for the analysis of cryo-preserved and formalin-fixed and paraffin-embedded (FFPE) tissue samples [13–17].

Intratumoral heterogeneity needs to be taken into account when assessing predictive or prognostic biomarkers from patient samples [18]. Our group previously demonstrated intratumoral variation in the expression of cell signaling proteins in high-grade serous ovarian carcinoma [15]. We addressed this challenge in the present study by combining multiple distinct regions of each tumor for analysis to avoid sampling bias.

The goal of this study was to identify differential expression of signaling pathway proteins associated with platinum resistance of ovarian cancer. To our knowledge, this is the first comprehensive characterization of signaling protein networks in platinum-sensitive versus platinum-resistant human ovarian cancer specimens.

## RESULTS

### Clinical characterization of tumor samples

A total of 192 patients with primary, non-recurrent advanced stage epithelial ovarian cancer diagnosed between 1999 and 2010 at a tertiary referral center, Klinikum rechts der Isar, Technische Universität München, Munich, Germany, were included. All patients underwent tumor debulking surgery followed by platinum-based adjuvant chemotherapy. *Of note, both platinum-sensitive and platinum-resistant tumor samples were obtained from non-recurrent previously untreated ovarian cancers*.

In order to allow for internal validation of our results, we applied a split-sample approach and patients were randomly allocated to either the training (*n* = 89) or validation cohort (*n* = 103). Both cohorts showed a similar distribution of patients’ ages, established clinico-pathologic parameters, as well as similar progression-free and overall survival. The median age was 61 (range 23 to 88) years, and the majority of patients had ovarian cancer of high-grade serous subtype (75%), followed by endometrioid subtype (11%). Most patients presented with advanced stages FIGO III and IV (84%) and about half of the patients (55%) had postoperative residual tumor. One third of the patients (29%) were platinum-resistant *defined by a progression-free interval of less than 6 months after completion of chemotherapy*. The majority of patients had received combination chemotherapy consisting of carboplatin plus paclitaxel (75%), while 13% had received platinum-monotherapy and 10% had received a third agent in addition to platinum- and taxane-based combination therapy. Patient characteristics are summarized in Table 1.

**Table 1:** Patient characteristics

	Total (*n* = 192)		Training set (*n* = 89)		Validation set (*n* = 103)	
	*n*	(%)	*n*	(%)	*n*	(%)
**Age, median (range) [years]**	61 (23; 88)		58 (23; 84)		61 (25; 88)	
Histologic subtype						
High-grade serous	144	(75%)	63	(71%)	81	(78%)
High-grade endometrioid	22	(11%)	15	(17%)	7	(7%)
Low-grade serous	3	(2%)	2	(2%)	1	(1%)
Mucinous	16	(8%)	8	(9%)	8	(8%)
Clear cell	7	(4%)	1	(1%)	6	(6%)
**FIGO Stage**						
I	23	(12%)	12	(13%)	11	(10%)
II	7	(4%)	4	(5%)	3	(3%)
III	147	(76%)	68	(76%)	79	(77%)
IV	15	(8%)	5	(6%)	10	(10%)
**Postoperative Residual Tumor**						
None	86	(45%)	41	(46%)	45	(44%)
Present	106	(55%)	48	(54%)	58	(56%)
**Platinum-Sensitivity**						
Sensitive	136	(71%)	69	(78%)	67	(65%)
Resistant	56	(29%)	20	(22%)	36	(35%)
**Chemotherapy Regimen**						
Carboplatin plus paclitaxel	144	(75%)	67	(75%)	77	(75%)
Carboplatin plus paclitaxel plus third agent^*^	20	(10%)	6	(7%)	14	(13%)
Carboplatin monotherapy	24	(13%)	13	(15%)	11	(11%)
Carboplatin plus cyclophosphamide	4	(2%)	3	(3%)	1	(1%)
**Follow-up, median, range [months]**	42 (1; 195)		41 (3; 167)		42 (1; 195)	
**Progression-free Survival**						
Median (95% CI) [months]	23 (18; 30)		27 (21; 46)		19 (14; 30)	
**Overall Survival**						
Median (95% CI) [months]	58 (48; 75)		65 (44; NA)		50 (42; 75)	
**Recurrence during follow-up**	140	(73%)	63	(71%)	77	(75%)
**Death during follow-up**	106	(55%)	46	(52%)	60	(58%)

Platinum sensitivity/ resistance, defined by a progression-free interval of ≥ / < 6 months after completion of chemotherapy

Progression-free and overall survival were calculated by Kaplan Meier method

^*^cyclophosphamide, epirubicine, gemcitabine, or topotecane; CI, confidence interval; NA, upper bound of 95% confidence interval not reached within follow-up period.

### Higher intratumoral PDGFRβ and VEGFR2 are associated with platinum resistance in epithelial ovarian cancer

A total of 11 signaling pathway proteins showed statistically significant differential expression between platinum-sensitive and platinum-resistant tumors in the training cohort (*n* = 89; *p* ≤ 0.05; Table 2). Of note, all proteins demonstrating differential expression were overexpressed in platinum-resistant versus platinum-sensitive ovarian cancers.

**Table 2:** Overexpression of signaling pathway proteins in platinum-resistant versus platinum-sensitive epithelial ovarian cancer

Protein	*p*-value for protein overexpression in platinum-resistant vs. platinum-sensitive	
	Training set (*n* = 89)	Validation set (*n* = 103)
Growth factors/receptors		
pEGFR (Tyr1086)	0.03^*^	0.98
HER2 (extracellular)	0.01^*^	0.59
HER3	0.03^*^	0.31
PDGFbb	0.03^*^	0.72
**PDGFRβ**	0.007^**^	0.04^*^
**VEGFR**2	0.02^*^	0.05^*^
pVEGFR2	0.03^*^	0.56
AKT-pathway		
mTOR	0.02^*^	0.41
PI3K	0.05^*^	0.40
MAPK-pathway		
JNK/SAPK	0.03^*^	0.47
ERK	0.008^**^	0.56

The table summarizes all signaling proteins showing statistically significant overexpression in platinum- resistant versus platinum-sensitive ovarian cancer in the training cohort. Proteins also showing statistically significant overexpression in the validation cohort are indicated in bold.

^*^ ≤ 0.05, ^**^ < 0.01.

The percentage of platinum-resistant patients was 22% (20/89) in the training set and 35% (36/103) in the validation set.

**Figure 1:**
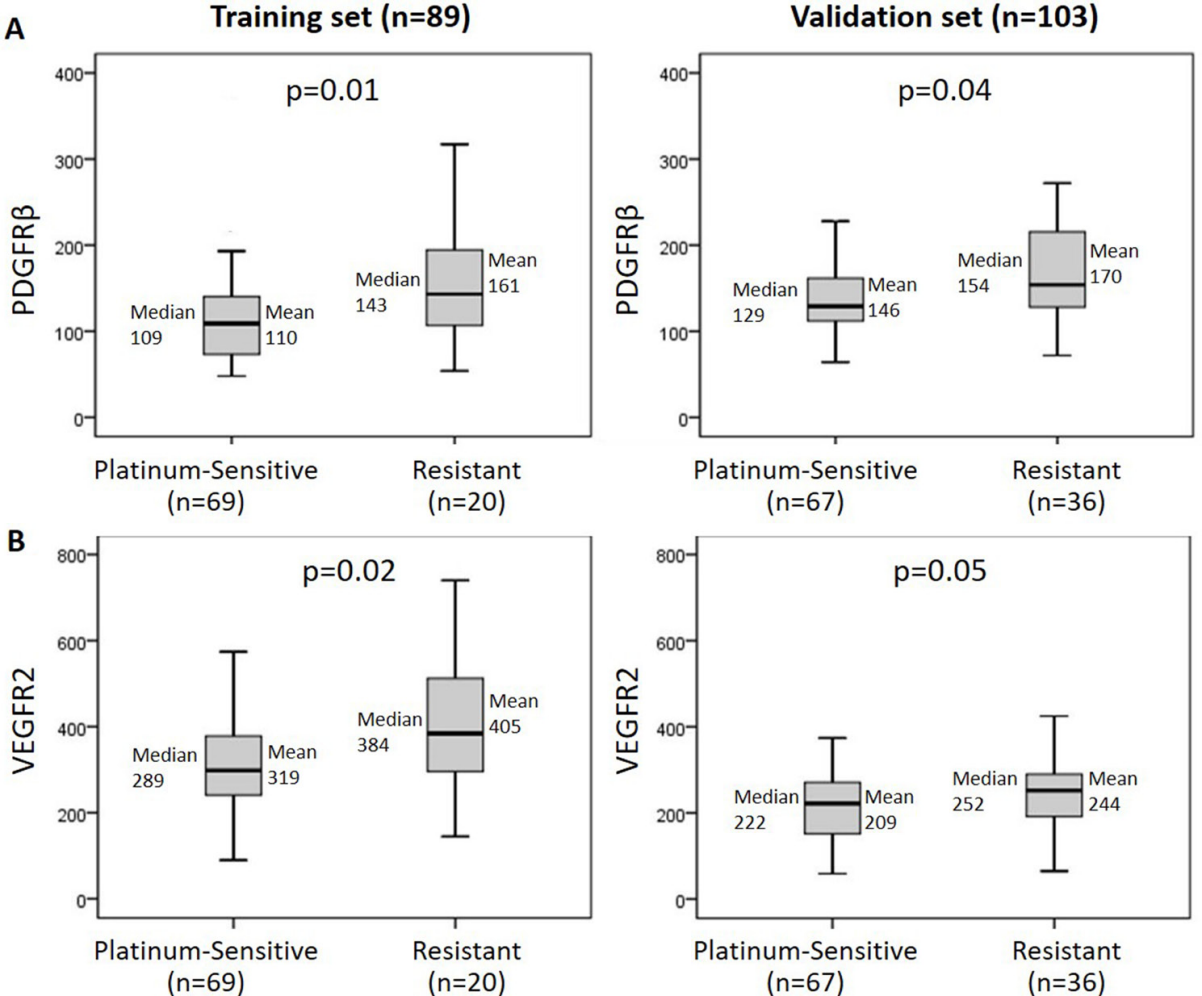
Increased PDGFRβ and VEGFR2 protein levels are associated with platinum resistance Higher intratumoral protein levels of PDGFRβ (**A**) and VEGFR2 (**B**) were demonstrated in platinum-resistant (defined as < 6 months progression-free interval following chemotherapy) vs. platinum-sensitive ovarian cancer patients in an independent training (*n* = 89) and validation cohort (*n* = 103).

Out of these 11 proteins the two growth factor receptors PDGFRβ and VEGFR2 also demonstrated significant overexpression in platinum-resistant tumors in the independent validation cohort (*n* = 103; Figure 1). Median PDGFRβ protein levels of platinum-resistant compared to platinum-sensitive tumors were 143 vs. 109 (relative units per total protein; *p* = 0.01) in the training cohort, and 154 vs. 129 in the validation cohort (*p* = 0.04). Median VEGFR2 protein levels were 384 vs. 289 (*p* = 0.02; training cohort) and 252 vs. 222 (*p* = 0.05; validation cohort) in platinum-resistant compared to platinum-sensitive tumors. The expression levels of PDGFRβ and VEGFR2 were correlated with each other with a Pearson correlation coefficient of 0.58 and 0.50 (*p* < 0.001) in the training and validation cohorts, respectively.

### Higher intratumoral PDGFRβ protein is associated with reduced survival

**Figure 2:**
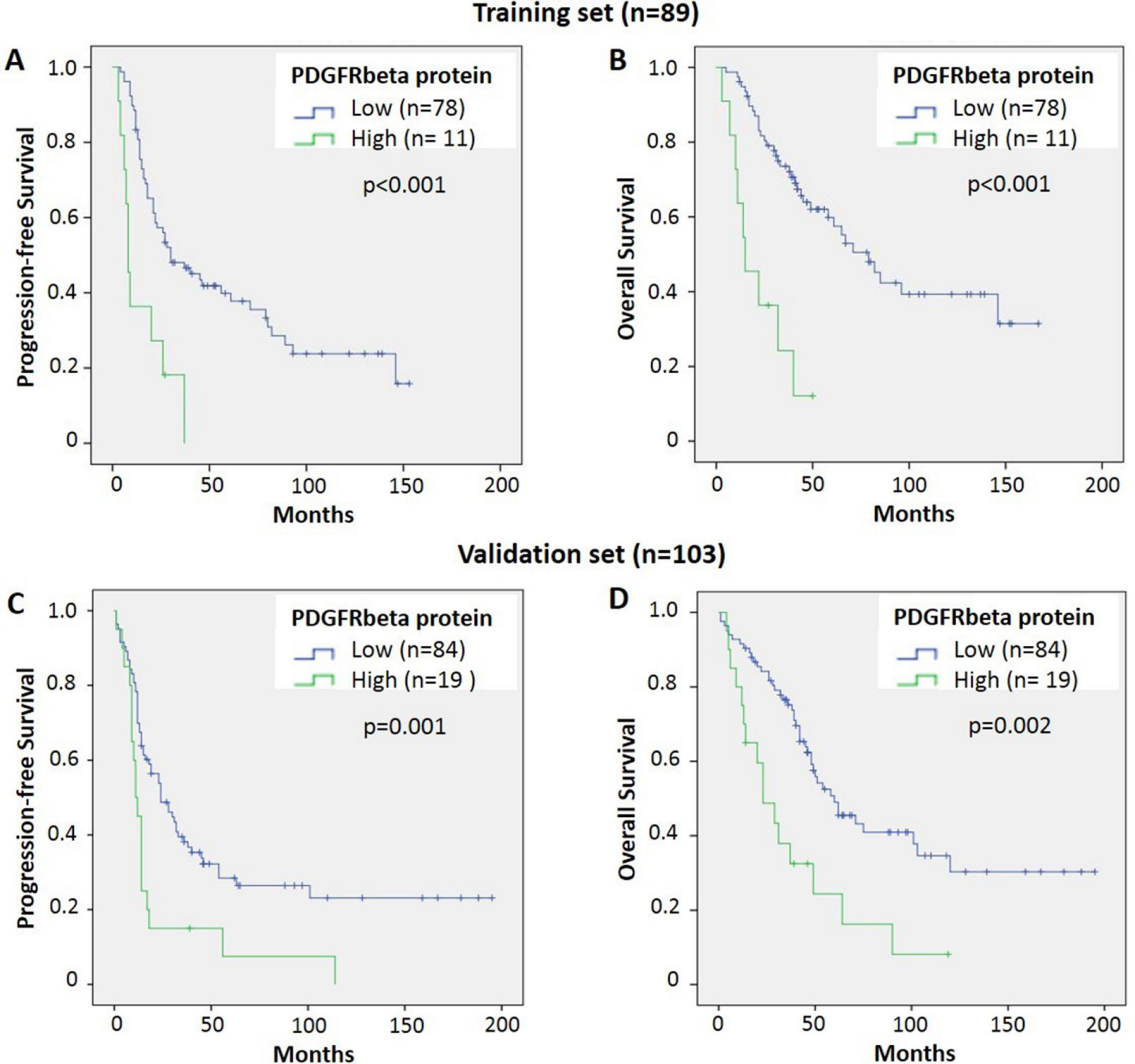
Higher PDGFRβ protein is associated with reduced survival of ovarian cancer patients Patients with higher intratumoral PDGFRβ (above 1.5^*^ median) demonstrated significantly shorter progression-free and overall survival (*p* < 0.01) compared to patients with low PDGFRβ (below 1.5^*^ median) in both training (**A**, **B**) and validation cohort (**C**, **D**).

Increased protein levels of PDGFRβ were significantly associated with shorter progression-free and overall survival in the training cohort (HR 2.4 and 3.6, respectively; *p* < 0.001) and validation cohort (HR 1.6 and 1.5, respectively; *p* = 0.03) (Table 3). Using a cutoff value of 1.5-times the median expression, eleven patients with high intratumoral PDGFRβ had a significantly shorter progression-free (median 8 vs. 30 months) and shorter overall survival (median 15 vs. 79 months) compared to 78 patients with low PDGFRβ (*p* < 0.001; Figure 2A, 2B) in the training cohort. In the validation cohort, 19 patients had high intratumoral PDGFRβ and demonstrated significantly shorter progression-free and overall survival compared to 84 patients with low PDGFRβ (median 12 vs. 29 months and 24 vs. 60 months, respectively; *p* < 0.01) (Figure 2C, 2D).

**Table 3:** Association between signaling pathway proteins and patient outcome

Protein	Training set (*n* = 89)				Validation set (*n* = 103)			
	OS		PFS		OS		PFS	
	[*p*]	HR	[*p*]	HR	[*p*]	HR	[*p*]	HR
Growth factors/ receptors								
**PDGFRβ**	< 0.001^***^	**3.6**	< 0.001^***^	**2.4**	0.03^*^	**1.6**	0.03^*^	**1.5**
pEGFR (Tyr1086)	0.07	2.4	0.36	1.5	0.97	1.0	0.84	1.0
pVEGFR2	0.09	2.2	0.35	1.5	0.13	1.8	0.36	1.3
AKT-pathway								
PTEN	0.08	1.5	0.19	1.3	0.26	1.1	0.22	1.1
MAPK-pathway								
ERK	0.004^**^	1.8	0.004^**^	1.8	0.34	1.1	0.62	1.1

The table summarizes all proteins showing an association with shorter overall or progression-free survival with a hazard ratio ≥ 1.3 (and *p*-value < 0.1) in the training cohort. Proteins also showing a significant association with survival in the validation cohort are indicated in bold.

^*^ ≤ 0.05, ^**^ < 0.01, ^***^ < 0.001.

HR, hazard ratio calculated for every increase in protein expression by 100 units.

OS, overall survival; PFS, progression-free survival.

In addition, we observed a statistically significant shorter overall survival with increasing protein levels of ERK (HR 1.8, *p* = 0.004) in the training cohort, with no considerable effect in the validation cohort (HR 1.1). The second protein strongly associated with platinum resistance, VEGFR2, showed no considerable association with overall survival in the training and validation cohorts (HR 1.1). All proteins associated with survival with a hazard ratio ≥ 1.3 are summarized in Table 3.

### Associations of signaling pathway proteins with established clinico-pathologic parameters

We next assessed associations between protein expression and common clinico-pathological tumor characteristics to identify potential confounding factors. We detected only infrequent associations of signaling pathway proteins with tumor stage or subtype. Out of 11 proteins, significantly higher levels of PI3K, ERK, and JNK/SAPK were detected in high-grade serous compared to other subtypes in the training cohort. In addition, advanced stage was associated with significantly higher levels of PI3K and ERK in the training cohort. None of these associations were observed in the validation cohort. None of the remaining proteins and neither PDGFRβ nor VEGFR2 were directly associated with any clinico-pathologic parameter.

### Advanced FIGO stage and postoperative residual tumor are associated with platinum resistance

To further assess potential confounding factors, we assessed possible associations between clinico-pathologic parameters and platinum resistance. Across the entire cohort (*n* = 192), patients with advanced stage (FIGO IIB-IV) were significantly more likely to be platinum-resistant compared to early stage (FIGO I-IIA) patients (34% vs. 4%, *p* = 0.001). Similarly, 44% (47/106) of patients with postoperative residual tumor were platinum-resistant compared to only 10% (9/86) of patients who had no residual tumor after surgery (*p* < 0.001). No considerable association was observed between tumor subtype and platinum resistance.

### Higher intratumoral levels of AKT-pathway related proteins and growth factor receptors are inconsistently associated with incomplete tumor resection

Increasing levels of several proteins were associated with a statistically significant higher likelihood for incomplete tumor resection (defined by the presence of postoperative residual tumor) in the training cohort, including ERK (OR 4.4, *p* = 0.002), extracellular HER2 (OR 3.3, *p* = 0.02), HER2 (OR 2.2, *p* = 0.05), and HER3 (OR 1.5, *p* = 0.04). However, no considerable or weak associations with resection status were observed in the validation cohort (OR ≤ 1.3, *p* > 0.1; Table 4).

**Table 4:** Increasing intratumoral levels of signaling pathway proteins associated with incomplete tumor resection

Protein	Training set (*n* = 89)		Validation set (*n* = 103)	
	[*p*]	OR	[*p*]	OR
Growth factors/ receptors				
VEGF	0.09	3.0	0.56	1.2
HER2 (extracellular)	0.02^*^	3.3	0.67	0.9
HER2	0.05^*^	2.2	0.73	1.1
HER3	0.04^*^	1.5	0.60	1.2
AKT-pathway				
PI3K	0.08	2.2	0.64	1.1
PTEN	0.08	1.8	0.28	1.2
MAPK-pathway				
ERK	0.002^**^	4.4	0.23	1.3

The table summarizes all proteins showing an association with incomplete tumor resection with an odds ratio ≥ 1.3 (and *p*-value < 0.1) in the training cohort.

^*^ ≤ 0.05, ^**^ < 0.01.

OR, odds ratio for incomplete tumor resection calculated for every increase in protein expression by 100 units.

### Higher PDGFRβ and VEGFR2 gene expression is associated with poor patient outcome in publicly available microarray datasets

**Figure 3:**
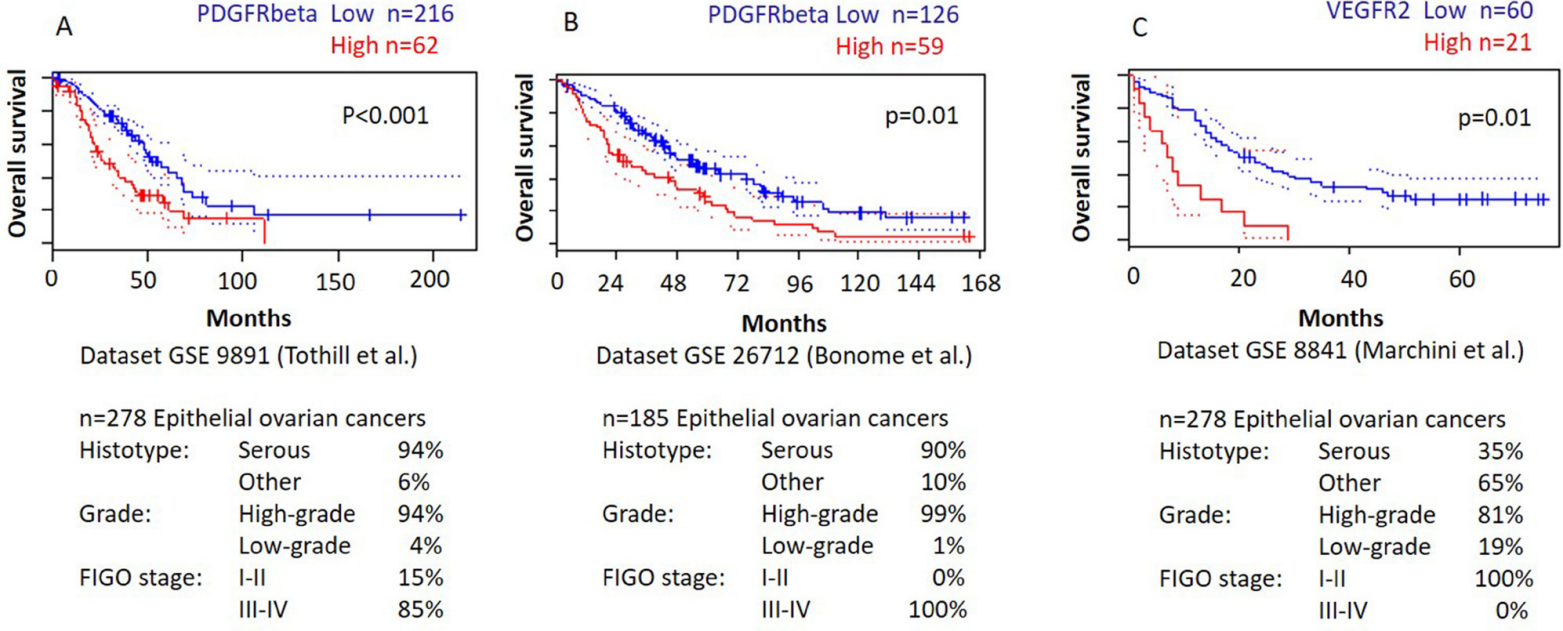
Higher PDGFRβ and VEGFR2 gene expression is associated with shorter overall survival in publicly available microarray datasets of ovarian cancer Patients with high PDGFRβ or VEGFR2 gene expression (red) showed significantly shorter overall survival compared to those with low PDGFRβ or VEGFR2 gene expression (blue) (*p* < 0.05). This Kaplan-Meier analysis was based on the PrognoScan database (http://www.prognoscan.org/) using three independent cohorts including 278 (**A**), 185 (**B**), and 85 (**C**) ovarian cancer patients, respectively, from the publicly available Gene Expression Omnibus (http://www.ncbi.nlm.nih.gov/geo) accession numbers GSE 9891 (A), GSE 26712 (B), and GSE 8841 (C) [20–22].

To further validate our findings, we analyzed the relationship between PDGFRβ and VEGFR2 mRNA expression levels and outcome of ovarian cancer patients using the PrognoScan database [19]. In three independent publicly available microarray datasets of ovarian cancer patients [20–22] we found a significant correlation between higher levels of PDGFRβ and VEGFR2 gene expression and reduced overall survival (Figure 3). Patient characteristics of these datasets are summarized in Supplementary Table 2.

## DISCUSSION

The common development of platinum resistance is a major determinant of poor outcome in advanced stage ovarian cancer [4]. Despite increased knowledge about drug resistance mechanisms [6, 23, 24], reliable biomarkers for prediction of platinum response or -resistance in ovarian cancer patients have been lacking. Our study demonstrates that two angiogenesis-related proteins, platelet derived growth factor receptor beta (PDGFRβ) and vascular endothelial growth factor receptor 2 (VEGFR2) are upregulated in platinum-resistant primary human ovarian cancer. High expression of these proteins in surgical specimens of untreated primary tumors was associated with platinum resistance, defined as relapse within 6 months after completion of chemotherapy, and shorter overall survival.

Increased levels of VEGFR have previously been associated with ovarian cancer progression and malignant ascites [25]. PDGFRβ expression is found in the majority of serous ovarian carcinomas, in particular high-grade serous cancers [26]. Increased levels of the second PDGFR isoform, PDGFRα was associated with shorter overall survival of 176 ovarian cancer patients [27]. Our results suggest that VEGFR2 and PDGFRβ could be useful to identify platinum-resistant tumors prior to first-line systemic therapy. The predictive value of high intratumoral PDGFRβ and VEGFR2 protein expression was independent of other clinico-pathologic parameters such as tumor stage or histologic subtype.

Only high intratumoral PDGFRβ was associated with significantly shorter median progression-free (8 vs. 30 months) and overall survival (15 vs. 79 months) in both the training and validation cohorts, respectively. In contrast, VEGFR2 showed no considerable association with overall survival (HR 1.1) despite the association with platinum resistance.

The prognostic value of both PDGFRβ and VEGFR2 was confirmed at the mRNA level by our analysis of three independent publicly available microarray datasets, demonstrating a significant correlation between higher PDGFRβ and VEGFR2 gene expression and reduced overall survival of ovarian cancer patients [19–22].

The majority of patients with epithelial ovarian cancer currently receive anti-angiogenic treatment in addition to platinum-based chemotherapy, frequently with the humanized anti-VEGF antibody bevacizumab. While the addition of bevacizumab to chemotherapy has improved progression-free survival of primary and recurrent ovarian cancer, this approach failed to increase overall survival or cure rates, and is associated with significant cost and side effects, requiring predictive markers for patient selection [28, 29].

PDGFRβ and VEGFR2 predicted resistance to platinum-based chemotherapy and might potentially aid in the selection of patients for anti-angiogenic therapies. Beyond anti-VEGF antibodies, several tyrosine kinase inhibitors targeting VEGF- and PDGF-receptors such as sunitinib, sorafenib, and pazopanib are currently under clinical investigation in recurrent ovarian cancer [30]. Pazopanib is an oral tyrosine kinase inhibitor targeting VEGF receptors-1, -2 and -3, PDGF receptor-α and -β and c-kit, resulting in the inhibition of angiogenesis and tumor proliferation [31]. A recent Phase III trial demonstrated improved progression-free survival for advanced ovarian cancer patients who received pazopanib as a maintenance therapy following primary surgery and first-line platinum-based chemotherapy [32]. However, pazopanib was associated with grade 3 and 4 toxicity requiring treatment discontinuation in 33% of patients [32].

Derived from our findings high protein levels of PDGFRβ and VEGFR2 in primary untreated tumor specimens might help to identify ovarian cancer patients in whom platinum resistance and the potentially improved efficacy of PDGFR- and/or VEGFR-inhibition may balance toxicities and side effects of anti-angiogenic treatment. A clinical trial investigating the addition of anti-angiogenic treatment on the outcome of patients with high levels of PDGFRβ and VEGFR2 would be highly desirable.

The need for an accurate selection of patients undergoing anti-angiogenic therapy is emphasized by the lack of a benefit in patients with an ‘immune activated subtype’ of ovarian cancer identified by gene expression profiling [33]. Recent *in-vitro* and *in-vivo* studies have demonstrated that VEGF/VEGFR pathways are involved in the differentiation and function of immune cells [34, 35]. High VEGF expression of ovarian cancers was associated with induction of myeloid derived suppressor cells, inhibiting local anti-tumor immunity in-*vivo* and contributing to poor prognosis [34].

Limitations of our study include the retrospective nature and limited number of patients (*n* = 192). We aimed to address this by defining an independent training (*n* = 89) and validation set (*n* = 103). Our tissue specimens were from a homogenous patient cohort including only newly diagnosed advanced stage primary tumors from previously untreated epithelial ovarian cancer. Both PDGFRβ and VEGFR2 are expressed in epithelial tumor cells as well as stromal and endothelial cells. Therefore, all tumor samples were microdissected to ensure high tumor cell content (>70%) for protein analysis. Tissue was obtained from two or more distinct locations of each individual tumor specimen to account for intratumoral heterogeneity and to avoid sampling bias.

In conclusion, we demonstrated that platinum-resistant ovarian cancers are characterized by distinct upregulations of PDGFRβ and VEGFR2. High intratumoral levels of these angiogenesis-related growth factor receptors might serve as novel predictive biomarkers to identify primary resistance to platinum-based chemotherapy. Those ovarian cancer patients with high expression of PDGFRβ and VEGFR2 might particularly benefit from additional anti-vascular therapy such as anti-VEGF antibodies or novel tyrosine kinase inhibitors targeting PDGFR and VEGFR. Future prospective studies are needed to investigate the predictive value of PDGFRβ and VEGFR2 for response to novel anti-angiogenic agents alone or in combination with chemotherapy.

## MATERIALS AND METHODS

### Patient samples

Patients with primary non-recurrent epithelial ovarian cancer diagnosed between 1999 and 2010 at a tertiary referral center, Klinikum rechts der Isar, Technische Universität München, Munich, Germany, were included. Further inclusion criteria were primary tumor debulking surgery, and an adjuvant chemotherapy regimen including carboplatin as a single agent or in combination with paclitaxel, with or without addition of a third chemotherapeutic agent (cyclophosphamide, epirubicine, gemcitabine, or topotecane). The study protocol was approved by the institutional review board and written informed consent was obtained from all patients.

Platinum-sensitive as well as platinum-resistant tumor samples were obtained from non-recurrent previously untreated ovarian cancers. Only primary tumor samples were analyzed in this study, excluding peritoneal, omental and lymph node metastases. Surgical specimens were formalin fixed and paraffin embedded according to standard procedures. H&E stained sections of all cases were reviewed by a pathologist (SA, MB) to confirm histologic subtype and assess the percentage of viable invasive tumor cells. Ovarian cancer samples were further processed if at least one well circumscribed area with a tumor cellularity > 70% was present to allow for manual microdissection. Of note, for all cases tumor tissue from two or more different locations of each primary tumor was combined to account for intratumoral heterogeneity and avoid or limit sampling bias.

### Protein extraction

All tissue samples from the same patient were processed simultaneously. Protein extraction was performed as previously described [16]. Briefly, FFPE tissue sections were deparaffinized, and proteins were extracted using EXB Plus buffer and heat treatment (Qiagen, Hilden, Germany). 2–7 sections of 10 μm thickness were processed in 100–170 μl of extraction buffer. The Bradford protein assay (BioRad, Hercules, US) was used according to the manufacturer's instructions to determine protein concentrations. Protein concentrations were adjusted to 2mg/ml with EXB Plus buffer. A Western blot probing for β-actin was performed from randomly selected lysates (*n* = 11) to demonstrate successful protein extraction and suitability for reverse phase protein array analysis. All protein lysates produced a clear β-actin band on Western blot.

### Analysis of protein expression by reverse phase protein arrays (RPPA)

The expression levels of 41 signal transduction proteins including 16 phosphorylated proteins were determined by RPPA. Antibodies and experimental conditions are summarized in Supplementary Table 1. RPPAs were generated using the SpotBot^®^ Extreme Microarray Spotter according to the manufacturer's instructions (Arrayit, Sunnyvale, CA 94089, USA). For every lysate and dilution (adjusted (2 mg/ml), 1:2, 1:4, 1:8, 1:16, extraction buffer), 2 replicates were applied onto a nitrocellulose coated glass slide (Grace Bio-Labs, Bend, US), which produced 12 data points per sample.

All samples of the training (*n* = 89) and validation cohort (*n* = 103), respectively, were spotted onto a single glass slide and processed simultaneously to avoid bias. Immunodetection was performed similar to Western blot analysis as previously described [36]. In brief, arrays were incubated with primary antibody at 4°C overnight, followed by incubation with secondary antibody for 1 hour at room temperature, and developed with enhanced chemiluminescence solution (4:1 mixture of ECL prime and ECL advanced; GE Healthcare, Buckinghamshire, UK). Exposure time was adjusted from five seconds to 30 minutes to achieve optimal signal intensity for subsequent quantification.

For estimation of total protein amounts, arrays were stained in parallel with Sypro Ruby Protein Blot Stain (Invitrogen, Karlsruhe, Germany) according to the manufacturer's instructions. Developed arrays were scanned and signal intensities quantified using standard curves derived from serial dilutions after background subtraction, and expression values were normalized to total protein content (MicroVigene software, Version 3.5.0.0, VigeneTech, Boston, USA).

Further details of the RPPA methodology, validation, and technical reproducibility have been previously described [16, 37]. All antibodies used in this study were validated for specificity by Western blot analysis.

### Statistical analysis

The Mann-Whitney *U* test was used to compare relative expression values of a given protein between groups of patients. The Chi-Square test was used to assess associations between clinico-pathologic parameters and platinum resistance. Cox-regression analysis was used to determine the hazard ratio and 95% confidence interval for death or recurrence in relation to protein expression levels. In addition, protein expression was used to classify patients into high and low expression using 1.5-times the median expression value as a cutoff. Kaplan Meier analysis was performed to calculate progression-free and overall survival and the log-rank test was used to compare survival probability between groups of patients. Binary logistic regression was utilized to assess the association between protein expression and surgical resection status (complete versus incomplete). Hazard ratio (HR) or odds ratio (OR) and 95% confidence intervals are shown for every increase in protein intensity by 100 units.

Box plots visualize differences in relative protein expression between groups of patients. Pearson correlation coefficient was used to assess bivariate relationship of quantitative parameters. All quantitative parameters are expressed as median and range or mean ± standard deviation (SD). All statistical tests were performed at a two-sided 5% level of significance using IBM SPSS Statistics (IBM Corporation, Version 21).

## SUPPLEMENTARY MATERIALS TABLES




