# Supplementary material for: Increased PDGFR-beta and VEGFR-2 protein levels are associated with resistance to platinum-based chemotherapy and adverse outcome of ovarian cancer patients
Source: Oncotarget. 2017 Jun 8;8(58):97851–61. doi: 10.18632/oncotarget.18415 (PMC5716696; doi:10.18632/oncotarget.18415)
Supplement: Supplementary file 1 [file oncotarget-08-97851-s001.pdf]

Increased PDGFR-beta and VEGFR-2 protein levels are associated with resistance to platinum-based chemotherapy and adverse outcome of ovarian cancer patients

Supplementary Materials

Supplementary Table 1: (A) Primary and (B) secondary antibodies used for RPPA and western blot analysis. See Supplementary_Table_1.

Supplementary Table 2: Characteristics of external patient cohorts used in PrognoScan analysis

	Tothill et al. (*n* = 267*)		Bonome et al. (*n* = 185)		Marchini et al. (*n* = 68*)	
	n	**(%)**	***n***	**(%)**	***n***	**(%)**
**Years of diagnosis included**	1992–2006		1990–2003		1992–2003	
**Age, median (range) [years]**	59 (23; 80)		64 (26; 85)		52 (n/a)	
**Histologic subtype**						
Serous	246	(92%)	166	(90%)	24	(35%)
Endometrioid	20	(8%)	0		17	(25%)
Mucinous	0		0		10	(15%)
Clear cell	0		0		16	(24%)
Other/ unknown	1	(< 0.5%)	19	(10%)	1	(1%)
**Grade**						
High-grade / grade 2–3	252	(94%)	184	(99%)	55	(81%)
Low-grade / grade 1	11	(4%)	1	(1%)	13	(19%)
unknown	4	(2%)	0		0	
**FIGO Stage**						
I	16	(6%)	0		68	(100%)
II	14	(5%)	0		0	
III	212	(79%)	144	(78%)	0	
IV	21	(8%)	41	(22%)	0	
unknown	4	(2%)	0		0	
**Postoperative Residual Tumor**						
None	68	(26%)	n/a		n/a	
≤ 1 cm	76	(28%)	90	(49%)	n/a	
> 1 cm	70	(26%)	95	(51%)	n/a	
**Chemotherapy Regimen**						
Platinum-based	243	(91%)	185	(100%)	43	(63%)
None	22	(8%)	0		25	(37%)
Other	2	(1%)	0		0	
**Recurrence during follow-up**	74	(28%)	n/a		18	(27%)
**Death during follow-up**	111	(42%)	129	(70%)	13	(19%)

*The cohorts analyzed by PrognoScan included 18 (Tothill et al.) and 15 (Marchini et al.) additional ovarian borderline tumors, respectively, which are not included in patient characteristics.

No data was available regarding platinum-sensitivity, median progression-free and overall survival, or median follow-up time of these patient cohorts.
