# Supplementary material for: Increased PDGFR-beta and VEGFR-2 protein levels are associated with resistance to platinum-based chemotherapy and adverse outcome of ovarian cancer patients
Source: Oncotarget. 2017 Jun 8;8(58):97851–61. doi: 10.18632/oncotarget.18415 (PMC5716696; doi:10.18632/oncotarget.18415)
Supplement: Supplementary file 2 [file oncotarget-08-97851-s002.docx]

**Supplementary Table 1: (A) Primary and (B) secondary antibodies used for RPPA and Western Blot analysis**

| **Target protein** | **Molecular weight [kDa]** | **Reference number** | **Company** | **Dilution** | **dissolved in 5%** |
| --- | --- | --- | --- | --- | --- |
|  |  |  |  |  |  |
| **Growth factors and receptors** | |  |  |  |  |
| EGFR | 170 | #2232 | CST | 1:2000 | BSA |
| pEGFR (Tyr1086) | 170-180 | ZMD.504 | Invitrogen | 1:10000 | BSA |
| pEGFR (Tyr1148) | 170 | #4404 | CST | 1:1000 | MP |
| HER2 | 185 | AO485 | Dako | 1:1000 | TBST |
| HER2 (D8F12; extracellular) | 185 | #4290 | CST | 1:1000 | MP |
| pHER2 (Tyr1248) | 185 | BS4090 | BioWorld | 1:1000 | MP |
| HER3 | 185 | Ab40627 | Abcam | 1:2000 | MP |
| pHER3 (Tyr1289) | 185 | #4791 | CST | 1:200 | BSA |
|  |  |  |  |  |  |
| **AKT pathway molecules** | | | | | |
| AKT | 60 | #9272 | CST | 1:1000 | BSA |
| pAKT (Ser473) | 60 | #4060 | CST | 1:500 | BSA |
| FAK | 125 | #3285 | CST | 1:3000 | MP |
| GSK-3β | 46 | #9315 | CST | 1:1000 | BSA |
| pGSK-3β (Ser9) | 46 | #9336 | CST | 1:1000 | BSA |
| mTOR | 298 | #4517 | CST | 1:2000 | MP |
| PI3K | 85 | #4292 | CST | 1:1000 | MP |
| pPI3K (p85 Tyr458, p55 Tyr199) | 60, 85 | #4228 | CST | 1:500 | MP |
| PRAS40 | 40 | #2691 | CST | 1:1000 | BSA |
| pPRAS40 | 40 | #2997 | CST | 1:1000 | BSA |
| PTEN | 54 | #9552 | CST | 1:2000 | MP |
| pPTEN | 54 | #9551 | CST | 1:2000 | BSA |
| 4EBP1 | 15-20 | #9644 | CST | 1:2000 | BSA |
| p4EBP1 (Tyr37/46) | 15-20 | #2855 | CST | 1:1000 | MP |
| S6RP | 32 | #2217 | CST | 1:5000 | BSA |
| pS6RP (Ser235/236) | 32 | #4858 | CST | 1:5000 | MP |
|  |  |  |  |  |  |
| **MAPK pathway molecules** | | | | | |
| JNK/SAPK | 46, 54 | #9252 | CST | 1:2000 | MP |
| p38 MAPK | 43 | #9212 | CST | 1:1000 | BSA |
| pp38 MAPK (Thr180/Tyr182) | 43 | #4631 | CST | 1:1000 | BSA |
| ERK (p44/42 MAPK) | 42, 44 | #9102 | CST | 1:1000 | BSA |
| pERK (Thr202/Tyr204) | 42, 44 | #9101 | CST | 1:1000 | BSA |
| B-Raf | 86 | #9434 | CST | 1:500 | MP |
| pB-Raf (Ser445) | 86 | #2696 | CST | 1:1000 | MP |
| PAK1 | 68 | #2602 | CST | 1:1000 | BSA |
|  |  |  |  |  |  |

| **Angiogenesis associated molecules** | |  |  |  |  |
| --- | --- | --- | --- | --- | --- |
| HIF-1α | 120 | #610959 | BD | 1:1000 | MP |
| VHL | 24 | #2738 | CST | 1:500 | MP |
| PDGF BB | 18, 29 | ab9704 | Abcam | 1:500 | MP |
| PDGFRβ (28E1) | 190 | #3169 | CST | 1:1000 | MP |
| pPDGFRβ (Tyr751) | 190 | #4549 | CST | 1:500 | BSA |
| VEGF | 24; 45 | ab46154 | Abcam | 1:1000 | MP |
| VEGFR2 | 210, 230 | 55B11 | CST | 1:500 (RPPA)  1:1000 (WB) | MP |
| pVEGFR2 (Tyr1175) | 230 | #2478 | CST | 1:500 | BSA |
|  |  |  |  |  |  |
| **Cell adhesion molecule** | |  |  |  |  |
| E-Cadherin | 120 | #610182 | BD | 1:5000 | MP |
|  |  |  |  |  |  |
| **Reference Proteins for Western Blot analysis** | | |  |  |  |
| β-Actin | 42 | A1978 AC-15 | Sigma-Aldrich | 1:10000 | MP |
| GAPDH | 37 | #2118 | CST | 1:2000 | MP |

All primary antibodies were stored at -20 °C, except HER2 (Dako) which was stored at 4 °C, and dissolved in 5 % skim milk powder (MP) or 5 % bovine serum albumin (BSA) solution.

Companies: Abcam (Cambridge, UK), BioWorld (Mt. Airy, USA), BD: Becton Dickinson (Franklin Lakes, USA*),* CST: Cell Signaling Technology Inc. (Danvers, USA), Dako (Hamburg, Deutschland), Invitrogen (Karlsruhe, Deutschland), Sigma-Aldrich (St.Louis, USA)

**B.**

| **Target protein** | **Stored at** | **Reference number** | **Company** | **Dilution** | **dissolved in 5%** |
| --- | --- | --- | --- | --- | --- |
| Anti-mouse-HRP | 4°C | NA931-1ML | GE HealthCare | 1:5000 | MP |
| Anti-rabbit-HRP | -20°C | #7074 | CST | 1:2000 | MP |

The secondary antibody anti-mouse-HRP was used for detection of the primary antibodies against mTOR, B-Raf, HIF-1α, β-Actin, and E-Cadherin. The remaining primary antibodies were detected with the secondary antibody anti-rabbit-HRP.
